# MiR-34b is associated with clinical outcome in triple-negative breast cancer patients

**DOI:** 10.1186/1746-1596-7-31

**Published:** 2012-03-22

**Authors:** Marek Svoboda, Jiri Sana, Martina Redova, Jiri Navratil, Marketa Palacova, Pavel Fabian, Ondrej Slaby, Rostislav Vyzula

**Affiliations:** 1 Department of Comprehensive Cancer Care, Masaryk Memorial Cancer Institute, Zluty kopec 7, Brno 656 53, the Czech Republic; 2 Department of Oncological and Experimental Pathology, Masaryk Memorial Cancer, Institute, Zluty kopec 7, Brno 656 53, the Czech Republic

**Keywords:** triple-negative breast cancer, miR-34a, miR-34b, miR-34c, prognosis

## Abstract

**Background:**

Breast cancer is the most common malignancy with the highest incidence rates among women worldwide. Triple-negative breast cancer (TNBC) represents the major phenotype of basal-like molecular subtype of breast cancer, characterized by higher incidence in young women and a very poor prognosis. MicroRNAs (miRNAs) are small non-coding RNAs playing significant role in the pathogenesis of many cancers including breast cancer. Therefore, miRNAs are also potential prognostic and/or predictive biomarkers in triple-negative breast cancer patients.

**Methods:**

Thirty-nine TNBC patients with available formalin-fixed paraffin-embedded (FFPE) tissues were enrolled in the study. MiR-34a, miR-34b, and miR-34c were analyzed using qRT-PCR and correlated to clinico-pathological features of TNBC patients.

**Results:**

Expression levels of miR-34b significantly correlate with disease free survival (DFS) (*p *= 0.0020, log-rank test) and overall survival (OS) (*p *= 0.0008, log-rank test) of TNBC patients. No other significant associations between miR-34a, miR-34b, and miR-34c with available clinical pathological data were observed.

**Conclusions:**

MiR-34b expression negatively correlates with disease free survival and overall survival in TNBC patients. Thus, miR-34b may present a new promising prognostic biomarker in TNBC patients, but independent validations are necessary.

## Background

Breast cancer is the most common malignancy with the highest incidence rates among women worldwide. It is a heterogeneous disease due to complicated etiology involving both genetic and environmental factors. This malignity comprises multiple entities associated with distinctive histological and biological features, clinical presentations and behaviours and responses to therapy. From the molecular point of view, breast cancers can be divided into six molecular subtypes [1,2]. Breast cancer with triple-negative phenotype (TNBC) characterized by the absence of the estrogen receptor alpha (ERα), the progesterone receptor (PgR), and the human epidermal growth factor receptor-2 (HER2) expression represents the major phenotype associated with basal-like molecular subtype of breast cancer. Additional molecular alterations connected with TNBC are the loss of function of BRCA1 gene (>11%), and a higher rate of p53 mutations (>82%). Both are significantly associated not only with breast cancer risk but also have been shown to negatively affect clinical outcome of breast cancer patients [3-5]. TNBC accounts for about 11% - 20% of all breast cancers. It is diagnosed more frequently in younger and premenopausal women and patients with TNBC who have significantly increased risk of relapse and death [3,4,6,7]. Because of a poor survival of TNBC patients, there is a clinical need to identify new prognostic biomarkers that can be used to predict a therapeutic response and clinical outcomes in TNBC patients to rationalize treatment decisions.

MicroRNAs (miRNAs) are highly conserved, small, non-coding RNAs, 18-25 nucleotides in length, that act as posttranscriptional regulators of gene expressions by silencing their mRNA targets. Recent studies showed that miRNAs regulate a significant number of oncogenes, tumor suppressor genes, and genes associated with the invasion, dissemination, and chemoresistance of tumors. Therefore, these molecules play significant roles in the pathogenesis of many cancers, including the breast cancer and its triple-negative subtype [8,9]. Many recent works described that the gene product of p53 which shows a high rate of mutations in TNBC patients, activates transcription of the set of miRNAs including the miR-34 family. The miR-34 family regulates cell cycle progression, cellular senescence and apoptosis and, as a consequence, could affect biological features of TNBC [10-12]. The aim of this study was to quantify expression levels of miR-34a, miR-34b, and miR-34c in the clinical samples of TNBC, and to evaluate their association with clinical- pathological features to identify new prognostic and/or predictive biomarkers for TNBC patients.

## Materials and methods

### Patients and samples

The study included a group of 39 clinical-pathological characterized patients with histologically confirmed triple-negative breast cancer (TNBC) (Table 1). All patients were females diagnosed with an early breast cancer and were treated according to the best clinical practices and protocols valid at the time of diagnosis. All patients complied with the designed treatment (except 3 women who refused the adjuvant therapy). A relapse of the disease was documented in 27 (69%) patients. Median follow-up time was 34 months for all and 124 months for living patients. In the whole group, the median DFS was16 months (range from 3.5 to 187 months) and median OS was 41 months (range from 13 to187 months). Informed consent approved by the local Ethical Commission was obtained from each patient before the treatment.

**Table 1:** Characteristics of TNBC patients

	Total (n = 39)	%
**Age (years)**		

**Median (range)**	44 (29 - 75)	

**Grade**		

**I**	0	0

**II**	7	18

**III**	32	82

**Histology**		

**Medullary**	6	15

**Invasive ductal**	33	85

**BRCA1 status**		

**Wild-type**	8	21

**Mutated**	13	33

**NA**	18	46

**T**		

**1**	10	26

**2**	21	54

**3**	4	10

**4**	4	10

**N**		

**0**	17	44

**1**	13	33

**2**	7	18

**3**	2	5

**Clinical stage**		

**I**	7	18

**II**	21	54

**III**	11	28

**IV**	0	0

Annotation: NA - not available

All samples were tissues collected surgically under the supervision of an experienced pathologist. Immunohistochemistry (IHC) evaluation was performed on formalin-fixed paraffin-embedded (FFPE) samples. HER2 negativity was defined as either the nonexpression of HER2 protein (IHC score 0/1+) or normal HER2 gene status. The expression of HER2 protein was determined by DAKO Herceptest (DAKO, Sweden) and scored on a qualitative scale from 0 to 3+ according to DAKO manual and American Society of Clinical Oncology/College of American Pathologists guideline recommendations for human epidermal growth factor receptor 2 testing in breast cancer. HER2 gene status was evaluated by FISH method using Abbott PathVysion HER2 kit (Abbott Laboratories, USA). HER2 gene status was considered as negative (FISH non-amplified) in case where a HER2 gene/centromer of chromosome 17 ratio was less than 1.8. All tumors were IHC 0 and/or FISH non-amplified. The estrogen receptor alpha (ERα) and progesterone receptor (PgR) status was examined by IHC using antibodies provided by Lab Vision (SP1 resp. SP2 monoclonal rabbit antibody, Lab Vision Thermo Fisher Scientific, USA). ERα and PgR status was considered positive if more than 10% of cells were stained in cell nuclei. However, all carcinomas included in our study did not have any expression of ERα and PgR (i.e. 0%). Clinical data were reviewed retrospectively from medical records. Response to therapy was evaluated by using RECIST criteria version 1.1.

### MiRNA extraction

Small RNA-enriched total RNA was isolated from FFPE samples using mirVana miRNA Isolation Kit (Ambion, USA). Nucleic acid concentration and purity were controlled by UV spectrophotometry (A260:A280 > 2.0; A260:A230 > 1.8) using Nanodrop ND-1000 (Thermo Scientific, USA).

### Real-time quantification of miRNAs by stem-loop RT PCR

Complementary DNA (cDNA) was synthesized from 10 ng small RNA-enriched total RNA using gene-specific primers and Taq-Man MicroRNA Reverse Transcription kit according to the Taq-Man MicroRNA Assay protocol (Applied Biosystems, USA). Real-Time PCR was performed using the Applied Biosystems 7500 Real-Time PCR System in accordance with the TaqMan MicroRNA Assay protocol. Reverse transcription as well as Real-Time PCR detection were carried out using hsa-miR-34a, hsa-miR-34b, and hsa-miR-34c (Assay no. 4427975; Applied Biosystems, USA). RNU6B (Assay no. 4427975; Applied Biosystems, USA) was used as a reference gene. The threshold cycle data were determined using the default threshold settings.

### Statistical analysis

Statistical analysis of differences of miRNA expression levels in TNBC groups that were divided on the basis of clinical-pathological features were evaluated using the Kruskall-Wallis test. Survival analyses were performed by Kaplan-Meier method and the differences between the survival curves were evaluated by the log-rank test to determine statistical significance levels. Variables significantly associated with DFS in univariate analyses were subjected to a stepwise backward multivariate analysis. P values <0.05 were considered significant. Multivariate analysis was performed according to the Cox's proportional hazards regression model. The MedCalc Version 11.4.2.0 (MedCalc Software, Belgium) was used for all calculations.

## Results

**Figure 1:**
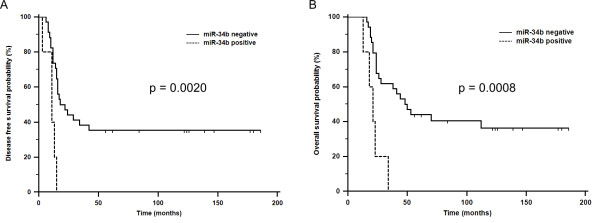
**Kaplan-Meier survival curves estimating disease free survival (A) and overall survival (B) of TNBC patients according to miR-34b expression levels**.

Survival analysis performed on the cohort of 39 TNBC patients showed that expression of miR-34b in tumors significantly correlates with a disease free survival (DFS) (P = 0.0020, log-rank test) (Figure 1a) and an overall survival (OS) (P = 0.0008, log-rank test) (Figure 1b) of TNBC patients. If relative expression levels of miR-34b were higher than zero, the patients were considered to be positive. Neither correlations with OS nor DFS were observed in the cases of miR-34a and miR-34c. Kruskal-Wallis analyses indicated no significant associations of miR-34a, miR-34b, and miR-34c with histology, BRCA1 status, clinical stage, grade, and regional lymph node metastases (Table 1).

## Discussion

TNBC is the most frequent phenotype associated with basal-like molecular subtype of breast cancer characterized mainly by ERα, PgR, and HER2 absence. Further typical molecular alterations of TNBC are the loss of function of BRCA1 gene and p53 mutations, both considered to be biomarkers of a breast cancer risk and a worse prognosis for breast cancer patients [4,5]. In this study, we have focused on the expression levels of miR-34 family whose members are transcriptionally activated mainly by p53. Majority of the previous studies described miR-34 family members as tumor suppressive miRNAs involved in the execution of p53-driven apoptotic pathways [10,12-15]. Our data show that miR-34b expression levels negatively correlate with a disease free survival (DFS) and an overall survival (OS) in TNBC patients, indicating rather oncogenic role in tumor cell. The similarly oncogenic feature of this miRNA was formerly described also in renal cell carcinomas and undifferentiated gastric cancers where higher expression levels of miR-34b in tumor tissues compared to non-tumor tissues were observed [16-18]. From the histopathological point of view, it is one of the features of TNBCs that is poorly differentiated and, thus, miR-34b expression could also be a marker of differentiation of the state of tumor cells [19]. This assumption is also supported by the fact that miR-34b is predicted (miRWalk Database) as a direct regulator of Notch2 which plays an important role in cell differentiation and is decreased in breast cancer tumors with poor differentiation (Grade 3) and a worse prognosis [20]. In breast cancer, Notch2 pathway is also a potent pro-apoptotic signaling with inhibitory effect on tumor growth in xenograft model [21]. Research group of Nevanlinna et al. (2011) revealed that overexpression of another member of miR-34 family, miR-34a, is a marker of an aggressive breast tumor phenotype (positive nodal status, ER-negativity, high proliferation rate, high grade, p53-positivity) [22]. Moreover, Dutta et al. has shown that knockdown of miR-34a significantly suppressed proliferation of MCF7 breast cancer cells [23]. These results together indicate that activation of miR-34 family could support proliferation, differentiation and aggressiveness of breast cancer cells. Statistical analyses have not shown any further significant association of miR-34a/b/c with the available clinical-pathological features. There is some evidence, however, that individual members of miR-34 family do not have to be expressed in the same rate; for example, in retinoblastoma cells or ovarian cancer tissues. These findings could be caused by p53-independent regulation of miR-34 family transcription that has been described in the case of miR-34a [24].

We have evaluated expression levels of miR-34 family in TNBC and came up with the following conclusion. The survival analyses have shown that the expression of miR-34b does negatively correlate with a disease free survival and an overall survival of TNBC patients. Therefore, we suggest that miR-34b could be a promising prognostic biomarker in TNBC patients. Our data are, however, preliminary and require other validations on a larger and independent group of TNBC patients.

## Abbreviations

TNBC: triple-negative breast cancer; ERα: estrogen receptor alpha; PgR: progesterone receptor; HER2: human epidermal growth factor receptor-2; BRCA1: breast cancer 1; OS: overall survival; DFS: disease-free survival.

## Competing interests

The authors declare that they have no competing interests.

## Authors' contributions

MS and OS designed the study, performed analysis and interpretation of data and the critical revision of the manuscript. JS, RM performed RNA purifications, qRT-PCR analysis, TLDA arrays and in vitro analysis. MS, JN, MP and RV collected clinical data of patients and controls involved in the study. MS performed statistical evaluation of the data. MS, JS and OS participated on the manuscript preparation. All authors read and approved the final manuscript.
